# Transition from child and adolescent mental health services to adult mental health services: children in care and adopted children

**DOI:** 10.1192/bjo.2021.466

**Published:** 2021-06-18

**Authors:** Paula Adamopoulos, Rani Samuel

**Affiliations:** 1 IoPNN King's College London; 2 Lewisham CAMHS

## Abstract

**Aims:**

Mental health transition-related disengagement is a major public health problem. This study aims to review children in care (CIC) and adopted children's transitions from child and adolescent mental health services (CAMHS) to adult mental health services (AMHS). This study aims to illustrate the often overlooked complexities that are associated with this population's transitions.

It is hypothesised that this population is at an increased risk for disengagement post-transition. Such is hypothesised as a result of the population's increased prevalence of complex mental health problems, neuro-developmental needs and developmental trauma. This population would benefit from a transition (optimal), as opposed to a transfer of care (suboptimal).

**Method:**

This retrospective case study included young people from Lewisham CAMHS's team for looked after and adopted children. Optimal transition was evaluated using four criteria: continuity of care, parallel care, a transition planning meeting and information transfer.

**Result:**

A total of 34 cases (male = 14, female = 20) were included, 88% of which were CIC (12% were adopted children). 85% of the cases included reports of at least one form of abuse and/or neglect. 59% of the cases were categorised as having more than one diagnostic group of mental health problems.

30% (n = 11) of the cases were discharged and were not recorded to have re-engaged with Lewisham AMHS. 12% of the cases had an outcome as 'unknown' due to miscellaneous reasons.

Only 18% (n = 6) of the cases had an ‘optimal’ transition. 18% (n = 6) had a suboptimal transfer and of those cases, 66% (n = 4) did not engage with AMHS beyond three months post-transfer. 21% (n = 7) were re-referred to Lewisham AMHS after being discharged from CAMHS. None of the re-referred cases engaged with AMHS post-referral.

**Conclusion:**

In conclusion, these findings demonstrate that this population is highly complex and can often experience suboptimal transitions from CAMHS to AMHS. Anything less than an 'optimal' transition yields a low ratel of therapeutic engagement. Recommendations for clinical practice includes an extended period of ‘overlap time’ between CAMHS to AMHS for CIC and adopted children. This overlap period will enable mental health practitioners to provide more informed and consistent support that incorporates the needs of CIC and adopted children. Such a provision will enhance therapeutic engagement and subsequently, promote better outcomes for CIC and adopted children. These findings have important resource implications for both CAMHS and AMHS teams.

